# Detection of Hazardous Liquids Using Microwaves

**DOI:** 10.6028/jres.119.009

**Published:** 2014-09-24

**Authors:** Michael D Janezic, Jolene D Splett, Kevin J Coakley

**Affiliations:** National Institute of Standards and Technology, Boulder, CO 80305

**Keywords:** binary classification, dielectric spectra, nearest neighbor, shielded-open coaxial fixture

## Abstract

We investigate the feasibility of using dielectric spectra to classify hazardous and nonhazardous liquids. The dielectric spectra of several liquids was obtained with a shielded-open coaxial fixture, and we present a new full-wave model for calculating the complex permittivity of liquids using this fixture. Using the measured complex permittivity for each liquid, we examine several classification methods for distinguishing between the hazardous and nonhazardous liquids and report on the error rates of each method.

## 1. Introduction

The primary objective of this study is to determine the feasibility of using low-power microwaves to distinguish between hazardous and non-hazardous liquids at security checkpoints. Our approach to liquid identification is based on the fact that the propagation of microwaves through liquids is substantially different than microwaves traveling in air. In general, both the frequency-dependent velocity and attenuation of microwaves will vary from liquid to liquid, depending on the liquid’s molecular composition, leading to the possibility of using this property to uniquely identify various liquids. However, instead of directly measuring the change in velocity and attenuation in this study, we measure a more fundamental quantity, the liquid’s complex permittivity, which we use as a basis for distinguishing one liquid from another.

In this report, we describe how we obtained accurate, broadband complex permittivity data, with the shielded-open coaxial measurement technique, for a number of hazardous and non-hazardous liquids, and then summarize the classification of these liquids using the complex permittivity as a basis for comparison. We conclude with a summary of this feasibility study and make some recommendations for the future research necessary to develop a screening method that can both quickly and accurately distinguish between hazardous and non-hazardous liquids.

## 2. Liquid Dielectric Spectra Measurements

**Fig. 1:**
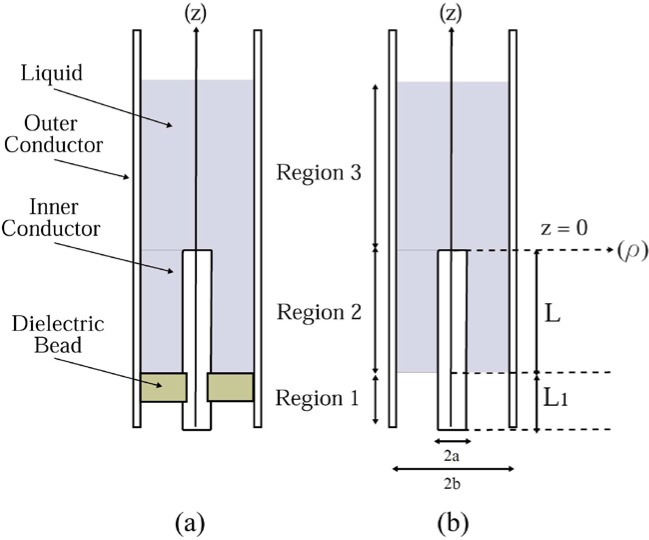
Cross-section of (a) shielded-open coaxial fixture and (b) simplified shielded-open coaxial fixture used in the modal-analysis model.

As mentioned above, the purpose of this study is to determine the feasibility of using microwave technology to identify dangerous liquids at a security checkpoint. The first step in this study is to determine if the liquids’ dielectric spectra exhibit sufficient contrast in the microwave region for this identification. To do this, we want to suppress as much as possible any effects that can obscure our data. Consequently, we used accurate, broadband, dielectric measurement techniques requiring advanced electromagnetic modeling, precisely-machined test fixtures, and high-frequency test equipment. Additional measurement challenges unique to liquids include containing the liquid within the measurement fixture and holding the liquid at a precise temperature, to avoid temperature-induced changes in the dielectric properties of the liquid under test. Because of all these factors, we chose the shielded-open coaxial fixture, shown in Fig. 1, as the measurement technique for this feasibility study. The next steps would be to migrate to continuously more realistic security checkpoint measurement systems and note the effects of this migration on system performance.

### 2.1 Shielded-Open Coaxial Fixture

The shielded-open coaxial fixture, shown in Fig. 1(a), was first employed as a high-frequency capacitance standard and eventually as an open standard for calibrating coaxial transmission lines with a vector network analyzer. Initial publications focused on developing models that accurately predicted the capacitance or the reflection coefficient of this device [1] from its physical dimensions.

Later, both Jesch [2] and Bussey [3] proposed the shielded-open coaxial fixture as a method for measuring the dielectric properties of liquids and soils at radio and microwave frequencies. By developing a model that related the shield-open fixture’s measured reflection coefficient to the complex permittivity of the dielectric material within the fixture, they were able to measure the dielectric properties of liquids and soils as a function of frequency.

A more accurate model for the shielded-open coaxial fixture was derived using modal analysis by Baker-Jarvis [4]. In this model, the complex permittivity of both solids and liquids could be measured, but because of the generality of this model (it must take into account both liquid and solids samples), the computational time necessary to compute the complex permittivity is significantly increased. In this report, we present a new model, also derived using modal analysis, that is specifically developed for measuring liquids using the shielded-open coaxial fixture, thus increasing the model’s computation speed while maintaining a high level of accuracy.

### 2.2 Mode-Matching Model for Shielded-Open Fixture

The shielded-open coaxial measurement fixture consists of three distinct regions, as shown in Fig. 1(a). Region 1 is a coaxial transmission-line section that is composed of an air-filled region and a region containing a low-loss dielectric bead. This bead provides the necessary mechanical support for the inner conductor as well as contain the liquid within the test fixture. To minimize the effect of any impedance mismatch created by the bead, the dimensions of both the bead and the inner conductor are chosen so that the characteristic impedance is approximately 50 Ω across both the airline and dielectric bead regions of region 1. Region 2 is also a coaxial transmission line, and the entire region between the inner and outer conductors is filled with the liquid under test. Region 3 is a circular-cylindrical waveguide section, whose diameter is the same as in regions 1 and 2. This region is completely filled with the liquid under test.

To simplify the model for the shielded-open fixture and improve the computational efficiency, we made several assumptions that reduce the fixture’s geometry to the approximation shown in Fig. 1(b). First, we replaced the combination air/bead in region 1, with a single, air-only region that has an effective length *L*_1_, an assumption that is valid as long as the dielectric bead is impedance matched. Next, if we operate at a frequency below the cut-off frequency of the cylindrical waveguide in region 3, the evanescent fields excited at the boundary between regions 2 and 3 will attenuate rapidly. In this case, for a sufficient amount of liquid, the fields do not extend all the way to the top of the liquid, and we can therefore assume that region 3 extends to infinity in the +*z* direction. Finally, we also assume that the inner and outer conductors are composed of perfect conductors. For most liquids, this simplification does not lead to significant errors in the calculation of the liquid’s complex permittivity. However, as we shall discuss later, this reduces our ability to measure the losses of low-loss liquids and may put a lower bound on measurement of the dielectric loss.

In order to perform permittivity measurements of liquids, the liquid-filled shielded-open coaxial fixture is connected to a calibrated vector network analyzer, and its reflection coefficient Γ_0_ is measured as a function of frequency. Using modal analysis, we derive a model for calculating a liquid’s broadband complex relative permittivity $\varepsilon_{s}^{*}=\varepsilon_{s}^{\prime }-j\varepsilon_{s}^{\prime \prime }$ from the measured reflection coefficient Γ_0_ at each frequency point.

#### 1) Tangential Fields in Region 1

We begin our analysis by defining the tangential electrical and magnetic fields in each of the three regions shown in Fig. 1. In region 1, we assume an that an incident TEM wave, originating from the vector network analyzer, travels in the positive (forward) *z* direction in the shielded-open coaxial fixture. At *z* = −*L*, the interface between regions 1 and 2, a portion of this wave is reflected back as a TEM mode due to the impedance mismatch created by the liquid in region 2. No evanescent *TM*_0_*_n_* modes are excited at the interface between regions 1 and 2 as the coaxial airline geometry is uniform across this boundary. Therefore, the tangential, time-harmonic electric and magnetic fields in region 1 are:

$$E_{\rho 1}(\rho ,z)=\underset{\text{incident TEM mode}}{\underset{︸}{R_{10}(\rho )e^{-\gamma_{10}(z+L)}}}+\underset{\text{reflected TEM mode}}{\underset{︸}{\Gamma_{0}R_{10}(\rho )e^{\gamma_{10}(z+L)}}}$$ (1)

and

$$H_{\phi 1}(\rho ,z)=\frac{j\omega \varepsilon_{0}}{\gamma_{10}}R_{10}(\rho )\left[e^{-\gamma_{10}(z+L)}-\Gamma_{0}e^{\gamma_{10}(z+L)}\right],$$ (2)

where Γ_0_ is the reflection coefficient, *R*_10_ is the radial eigenvector for the TEM mode, and $\gamma_{10}=j\sqrt{\omega^{2}\mu_{0}\varepsilon_{0}}$ is the propagation constant for the TEM mode.

From orthogonality of the eigenfunctions R_10_ and the fact that the longitudinal electric field goes to zero along the conductors of the coaxial line, we find that

$$R_{10}(\rho )=\frac{1}{\sqrt{\ln(b/a)}}\frac{1}{\rho },$$ (3)

where *a* is the radius of the inner conductor and *b* is the radius of the outer conductor.

#### 2) Tangential Fields in Region 2

In region 2 of the shielded-open coaxial fixture, the electric and magnetic fields are a superposition of forward and backward-traveling waves. Because of the impedance discontinuity at *z* = 0, we not only have a propagating TEM mode, but also evanescent TM_0_*_n_* modes. At this discontinuity (*z* = 0), therefore, we can define the tangential electrical and magnetic fields as

$$\begin{array}{l} E_{\rho 2}(\rho ,z)=A_{0}R_{20}(\rho )e^{-\gamma_{20}z}+B_{0}R_{20}(\rho )e^{\gamma_{20}z}\\ \;+\sum_{n=1}^{\infty }\left[A_{n}R_{2n}(\rho )e^{-\gamma_{2n}z}+B_{n}R_{2n}(\rho )e^{\gamma_{2n}z}\right] \end{array}$$ (4)

and

$$\begin{array}{l} H_{\phi 2}(\rho ,z)=\frac{j\omega \varepsilon_{0}\varepsilon_{s}^{*}}{\gamma_{20}}\left[A_{0}R_{20}(\rho )e^{-\gamma_{20}z}-B_{0}R_{20}(\rho )e^{\gamma_{20}z}\right]\\ \;+\sum_{n=1}^{\infty }\frac{j\omega \varepsilon_{0}\varepsilon_{s}^{*}}{\gamma_{2n}}\left[A_{n}R_{2n}(\rho )e^{-\gamma_{2n}z}-B_{n}R_{2n}(\rho )e^{\gamma_{2n}z}\right], \end{array}$$ (5)

where $\varepsilon_{s}^{*}=\varepsilon_{s}^{\prime }-j\varepsilon_{s}^{\prime \prime }$ is the complex relative permittivity of the liquid under test, $\gamma_{20}^{2}=-\omega^{2}\mu_{0}\varepsilon_{0}\varepsilon_{s}^{*}$ is the propagation constant of the TEM mode, and are $\gamma_{2n}^{2}=k_{2n}^{2}-\omega^{2}\mu_{0}\varepsilon_{0}\varepsilon_{s}^{*}$ the propagation constants of the evanescent TM_0_*_n_* modes.

At the lower boundary of region 2 (*z* = −*L*), we assume that the evanescent TM_0_*_n_* modes that were excited at *z* = 0 have attenuated sufficiently so that they can be neglected. Thus, at *z* = −*L*, the electrical and magnetic fields reduce to

$$E_{\rho 2}(\rho ,z)=A_{0}R_{20}(\rho )e^{-\gamma_{20}z}+B_{0}R_{20}(\rho )e^{\gamma_{20}z}$$ (6)

and

$$H_{\phi 2}(\rho ,z)=\frac{j\omega \varepsilon_{0}\varepsilon_{s}^{*}}{\gamma_{20}}\left[A_{0}R_{20}(\rho )e^{-\gamma_{20}z}-B_{0}R_{20}(\rho )e^{\gamma_{20}z}\;\right].$$ (7)

Because region 2 has the same cross-sectional dimensions as region 1, the radial eigenfunctions of the two regions are the same for the TEM mode

$$R_{20}(\rho )\equiv R_{10}(\rho )=\frac{1}{\sqrt{\ln(b/a)}}\frac{1}{\rho }.$$ (8)

Due to the presence of the TM_0_*_n_* modes in this region, we also have to determine the eigenfunctions *R*_2_*_n_* and eigenvalues *k*_2_*_n_* for the TM_0_*_n_* modes. Taking advantage of the orthogonality of the eigenfunctions *R*_2_*_n_* and the fact that the longitudinal electric field goes to zero along the conductors of the coaxial line, we find that

$$R_{2n}(\rho )=\frac{\pi k_{2n}}{\sqrt{2}}{\left[\frac{J_{0}^{2}(k_{2n}a)}{J_{0}^{2}(k_{2n}b)}-1\right]}^{-1/2}\times \left[J_{1}(k_{2n}\rho )Y_{0}(k_{2n}a)-J_{0}(k_{2n}a)Y_{1}(k_{2n}\rho )\right],$$ (9)

where *J*_0_ is the Bessel function of the first kind of order zero, *J*_1_ is the Bessel function of the first kind of order one, *Y*_0_ is the Bessel function of the second kind of order zero, and *Y*_1_ is the Bessel function of the second kind of order one. To find the values of the TM_0_*_n_* mode’s eigenfunctions *k*_2_*_n_*, we use the following transcendental equation

$$Y_{0}(k_{2n}a)J_{0}(k_{2n}b)-J_{0}(k_{2n}a)Y_{0}(k_{2n}b)=0.$$ (10)

#### 3) Tangential Fields in Region 3

Region 3 is a circular-cylindrical waveguide that is completely filled with the liquid under test. As there is no center conductor to support a TEM mode, only TM_0_*_n_* modes are present in this region. Also, since we operate at frequencies below the cut-off frequency of the waveguide, the TM_0_*_n_* modes are evanescent and will attenuate before they reach top of the liquid. Thus, we can assume that only forward-traveling TM_0_*_n_* modes exist in this region. The electric and magnetic fields in this region are:

$$E_{\rho 3}=\sum_{n=1}^{\infty }C_{n}R_{3n}(\rho )e^{-\gamma_{3n}z}$$ (11)

and

$$H_{\phi 3}=\sum_{n=1}^{\infty }\frac{j\omega \varepsilon_{0}\varepsilon_{s}^{*}}{\gamma_{3n}}C_{n}R_{3n}(\rho )e^{-\gamma_{3n}z},$$ (12)

where $\gamma_{3n}^{2}=k_{3n}^{2}-\omega^{2}\mu_{0}\varepsilon_{0}\varepsilon_{s}^{*}$. Due to the orthogonality of the radial eigenfunctions *R*_3_*_n_* and the boundary condition forcing the longitudinal electric field to zero along the waveguide walls, we find

$$R_{3n}(\rho )=\frac{\sqrt{2}}{bJ_{1}(k_{3n}b)}J_{1}(k_{3n}\rho ),$$ (13)

where the values of the eigenvalues *k*_3_*_n_* are determined from the following transcendental equation

$$J_{0}(k_{3n}b)=0.$$ (14)

#### 4) Matching Boundary Conditions

We have already satisfied the boundary conditions in the radial direction, but we must now match the remaining longitudinal boundary conditions in order to solve for the unknown coefficients *A_n_*, *B_n_*, *C_n_*, and Γ_0_. First, we enforce the boundary condition that the tangential electric and magnetic fields are continuous at *z* = −*L*

$$E_{\rho 1}(\rho ,z=-L)=E_{\rho 2}(\rho ,z=-L)$$ (15)

and

$$H_{\phi 1}(\rho ,z=-L)=H_{\phi 2}(\rho ,z=-L).$$ (16)

Substituting (6) and (7) into (15) and (16), we get the following two equations

$$\Gamma_{0}-A_{0}e^{\gamma_{20}L}-B_{0}e^{-\gamma_{20}L}=-1$$ (17)

and

$$\Gamma_{0}+\varepsilon_{s}^{*}\frac{\gamma_{10}}{\gamma_{20}}\left[A_{0}e^{\gamma_{20}L}-B_{0}e^{-\gamma_{20}L}\right]=1.$$ (18)

In a similar manner, we match the tangential electric and magnetic fields at the *z* = 0, the boundary between regions 2 and 3

$$E_{\rho 2}(\rho ,z=0)=E_{\rho 3}(\rho ,z=0)$$ (19)

and

$$H_{\phi 2}(\rho ,z=0)=H_{\phi 3}(\rho ,z=0)$$ (20)

to obtain

$$A_{0}R_{20}(\rho )+B_{0}R_{20}(\rho )+\sum_{n=1}^{N_{2}}B_{n}R_{2n}(\rho )=\sum_{n=1}^{N_{3}}C_{n}R_{3n}(\rho )$$ (21)

and

$$\frac{A_{0}R_{20}(\rho )}{\gamma_{20}}-\frac{B_{0}R_{20}(\rho )}{\gamma_{20}}-\sum_{n=1}^{N_{2}}\frac{B_{n}R_{2n}(\rho )}{\gamma_{2n}}=\sum_{n=1}^{N_{3}}\frac{C_{n}R_{3n}(\rho )}{\gamma_{3n}}.$$ (22)

In both (21) and (22), we have truncated the infinite series to a finite number of terms in regions 2 and 3. This approximation will have a negligible effect on the complex permittivity calculation provided we use enough terms to allow each series to converge. Further reduction of (21) and (22) is possible. If we multiply (21) by *ρR*_3_*_m_* (*ρ*) and integrate over [0, *b*], we get

$$(A_{0}+B_{0})<R_{20}R_{3m}>+\sum_{n=1}^{N_{2}}B_{n}<R_{2n}R_{3m}>-C_{m}=0.$$ (23)

In a similar manner, if we multiply (22) by *ρR*_2_*_m_* (*ρ*) and integrate over [*a*, *b*], we get

$$\frac{A_{0}\delta_{m0}}{\gamma_{20}}-\frac{B_{m}}{\gamma_{2m}}-\sum_{n=1}^{N_{3}}\frac{C_{n}}{\gamma_{3n}}<R_{2m}R_{3n}>=0,$$ (24)

where

$$<R_{20}R_{3m}>=\frac{\sqrt{2}}{bk_{3m}}\frac{1}{\sqrt{\ln\frac{b}{a}}}\frac{J_{0}(k_{3m}a)}{J_{1}(k_{3m}b)}$$ (25)

and

$$<R_{2m}R_{3n}>=\frac{2k_{3n}J_{0}(k_{3n}a)}{bJ_{1}(k_{3n}b)}{\left[\frac{J_{0}^{2}(k_{2m}a)}{J_{0}^{2}(k_{2m}b)}-1\right]}^{-\frac{1}{2}}\frac{1}{k_{3n}^{2}-k_{2m}^{2}}.$$ (26)

Equations (17), (18), (23), and (24) form a system of equations, whose *N*_2_ + *N*_3_ +1, unknowns are *A_n_*, *B_n_, C_n_* and the reflection coefficient Γ_0_. If we measure the reflection coefficient Γ_0_, we can iteratively solve for the liquid’s complex permittivity $\varepsilon_{s}^{\prime }$. We employed the Newton-Raphson technique to solve for the complex permittivity $\varepsilon_{s}^{\prime }$, and this technique requires an initial guess for the liquid’s complex permittivity.

### 2.3 Complex Permittivity Measurements

To measure the complex permittivity of various liquids over a frequency range of 10 MHz to 1 GHz, we fabricated a shielded-open coaxial fixture. The inner conductor’s diameter 2*a* is 3.04 mm, the outer conductor diameter 2*b* is 6.95 mm, and the coaxial inner conductor length *L* is 4.96 mm. The top of the fixture is terminated with a polytetrafluoroethylene cap to prevent any evaporation of the liquid during the measurement.

To calculate the complex permittivity of a liquid, we measured the reflection coefficient Γ_0_, from 10 MHz to 1 GHz, using a vector network analyzer. The network analyzer is first calibrated using a conventional open-short-load (OSL) calibration using APC-7 coaxial calibration standards. Once calibrated, the fixture is connected to the network analyzer through a APC-7 coaxial transmission line. In order to minimize the drift of the network analyzer, a calibration is performed prior to each measurement. Because the dielectric properties of liquids can be sensitive to temperature as well as frequency, the fixture is placed in an environmental chamber, where the temperature is held to 20 ± 0.1 °C during all of the measurements. Following each measurement, the fixture is fully disassembled and cleaned in an ultrasonic cleaner to prevent any cross contamination between consecutive measurements.

Once the liquid-filled measurement fixture stabilized at 20 °C in the environmental chamber, we measured the reflection coefficient Γ_0_ as a function of frequency with the vector network analyzer. Then, using the model outlined in Sec. 2.1, we calculated the liquid’s complex relative permittivity at each frequency point. For the first two validation studies, described in Sec. 3, we measured twenty liquids, shown in Table 1. To gain a better understanding of the measurement variation, we measured each liquid three times on three different days. This collection of liquids includes both hazardous and non-hazardous liquids that cover a large range of complex permittivity values.

**Fig. 2:**
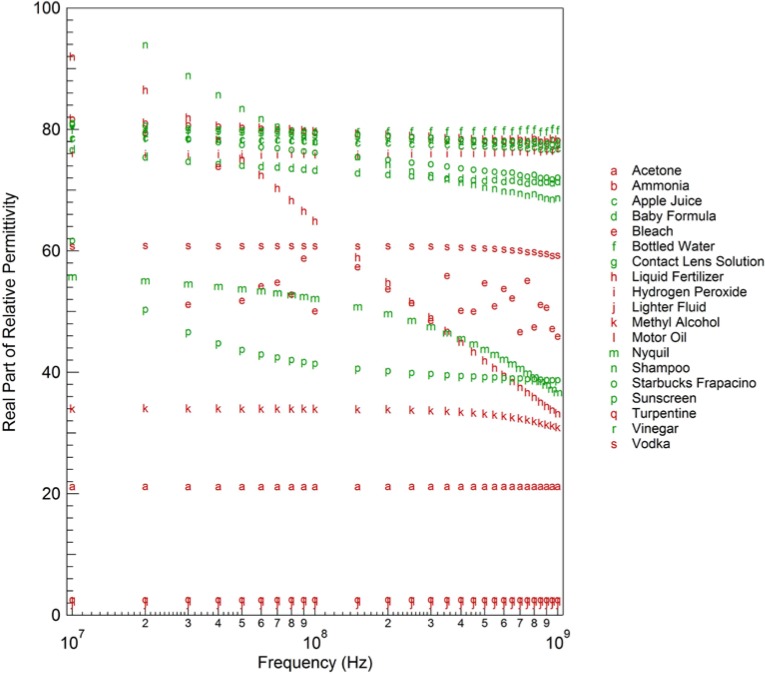
Real part of relative permittivity of various liquids at 20 C as a function of frequency.

**Fig. 3:**
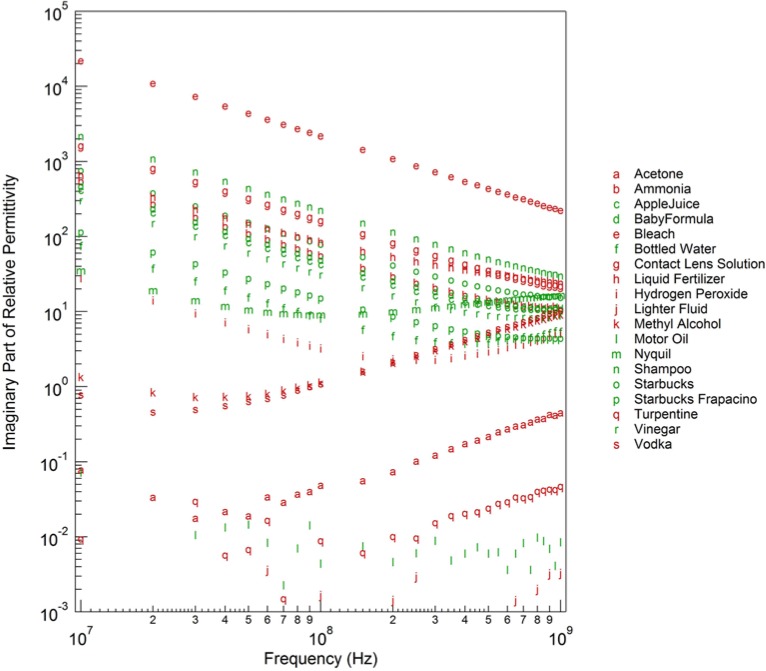
Imaginary part of relative permittivity of various liquids at 20 C as a function of frequency.

We show the real part of the relative permittivity $\varepsilon_{s}^{\prime }$ in Fig. 2 and the imaginary part of the relative permittivity $\varepsilon_{s}^{\prime \prime }$ in Fig. 3 for the first collection of twenty liquids. The real part of the relative permittivity varies from 2 for the low-loss, non-polar liquids (turpentine, motor oil, etc.) to values near 80, for the polar, water-based liquids (baby formula, contact lens solution, etc.), with many of the alcohols falling in between. The variation in the imaginary part of the relative permittivity is more dramatic, with the data spanning nine decades. This is mainly due to the variation in ion concentration, primarily salt content, that is present in many of the liquids. The higher the ion concentration, the larger the value of the imaginary part of the relative permittivity. These sixty dielectric spectra (twenty liquids measured three times) formed the foundation of our classification database.

In order to validate the classification models derived in the next section, we also measured ten additional “unknown” liquids, listed in Table 1, five of which were in the original study and five of which were new liquids. The complex permittivity of these “unknown” liquids was used to validate the classification models employed to determine whether the liquid was hazardous or not. Below, we describe the classification models and the validation study in some detail.

## 3. Classification Study and Validation

Since all liquids were measured at the same set of frequencies, we created a variable for each frequency to be used in various classification techniques. For example, if *ε*′ is measured at 28 different frequencies, then the classification analysis utilizes 28 variables with values that correspond to the *ε*′ measurements. Classification techniques were applied to seven representations of the data: *ε*′, *ε*″, magnitude, phase, loss tangent, combined *ε*′ and *ε*″, and combined magnitude and phase. Two responses were combined by simply including variables in the classification analysis for both responses. Preliminary studies indicate that the method with the best binary classification performance is the nearest neighbor method; thus all analyses and results presented in this document will pertain to the nearest neighbor method. Several other classification techniques were considered including: logistic regression, linear discriminant analysis, flexible discriminant analysis, neural networks, classification trees, and support vector machines. Information for all techniques can be found in [5]. Since a natural clustering (or separation) between hazardous and non-hazardous liquids is not readily apparent in our data, the traditional binary classification techniques were not very effective.

The nearest neighbor method can be thought of as a two-step process. First, we determine which spectrum in the database is closest to the unknown spectra based on Euclidean distance. Next, we classify the unknown spectra as being either hazardous or non-hazardous based on the identity of the nearest known spectra. For example, if the spectrum of an unknown liquid is closest to ammonia, then the unknown would be classified as being hazardous because ammonia is hazardous. If the spectrum of an unknown liquid is most similar to apple juice, then the unknown would be classified as non-hazardous because apple juice is non-hazardous. We used the indicator function,

$$I=\{\begin{array}{ll} 0 & non-hazardous\\ 1 & hazardous \end{array}$$ (27)

to assign a numerical value to the class of a liquid.

The nearest neighbor method was applied to each of seven representations of the measured data. We used a “majority vote” approach to combine the classification results for all responses to obtain an “overall” classification. In the majority voting process, a “score” is determined by adding indicator function values (*I*) observed for each response and then dividing by the total number of responses *n*. In our problem, the total number of responses is seven, where

$$\text{Score}=\frac{\sum_{i=1}^{n}I_{i}}{n}.$$ (28)

If the score is greater than 0.5, then the unknown liquid is classified as hazardous, otherwise the liquid is non-hazardous.

To evaluate the performance of the classification techniques, we conducted three validation studies, which are summarized below and in Table 2.

Validation Study 1: For each of the 60 spectra (20 liquids and three repeats per liquid) in the database, we excluded one spectrum and used the remaining 59 spectra to build a classification model. Then, we predicted the class (hazardous or non-hazardous) for the excluded spectrum. For this analysis, the database of 59 spectra contains two repeats of the liquid 7 under test.

Validation Study 2: For each liquid, we eliminated all three repeats and then used the remaining 57 spectra to build the classification model. Then we predicted the class for each of the three excluded spectra. For this analysis, the database of liquids does not contain the liquid under test.

Validation Study 3: Each of 10 unknown liquids in Table 1 are classified as being either hazardous or non-hazardous based on the classification model developed using the data from the primary experiment. Five of the unknown liquids are represented in the database and the remaining five are not.

### 3.1 Validation Studies 1 and 2

Table 3 displays error rates as well as the true positive and false positive error rates for Validation Studies 1 and 2 based on the nearest neighbor classification of liquids (hazardous, non-hazardous) for all responses and the majority vote. The true positive error rate is the proportion of cases in which a liquid is correctly classified as hazardous given that the liquid is hazardous. The false positive error rate is the proportion of cases in which a liquid is incorrectly classified as hazardous given that the liquid is nonhazardous.

The results shown in Table 3 indicate that if an unknown liquid is contained in the database, as in Validation Study 1, we have a good chance of detecting a hazardous liquid using the nearest neighbor method based on the magnitude or the combined real and imaginary parts of permittivity. However, the classification error rates associated with Validation Study 2, in which the liquid is not contained in the database, are very high regardless of the response. For Validation Study 1, a hazardous liquid is always classified correctly, except for two cases using *ε*′, and the probability of obtaining a false positive is quite small. However, the probability of correctly classifying a hazardous liquid is low, and the probability of obtaining a false positive is very high for Validation Study 2.

### 3.2 Validation Study 3 Results

Next, we used the 20 liquids in our database to predict the class of ten unknown liquids with the nearest neighbor method for each of the seven responses. Table 4, displays the class (hazardous/non-hazardous) of the unknown liquids based on the nearest neighbor for each of the responses, the class and score determined by majority vote, and the true class of the unknown. All the unknown liquids were correctly classified for all responses except for *ε*′ and the combined *ε*′ and ε″. The two liquids incorrectly classified using *ε*′ were contact solution, which was contained in the database, and orange juice, which was not. For the combined *ε*′ and *ε*″ response, we incorrectly classified orange juice as being hazardous. Based on the majority vote, each of the ten unknown liquids was matched to a liquid from the database of the same class.

**Fig. 4:**
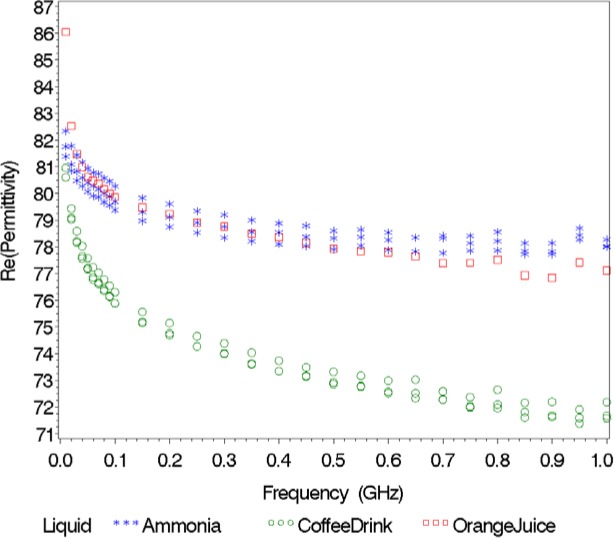
Real part of relative permittivity versus frequency for orange juice (the unknown) and its nearest neighbors.

**Fig. 5:**
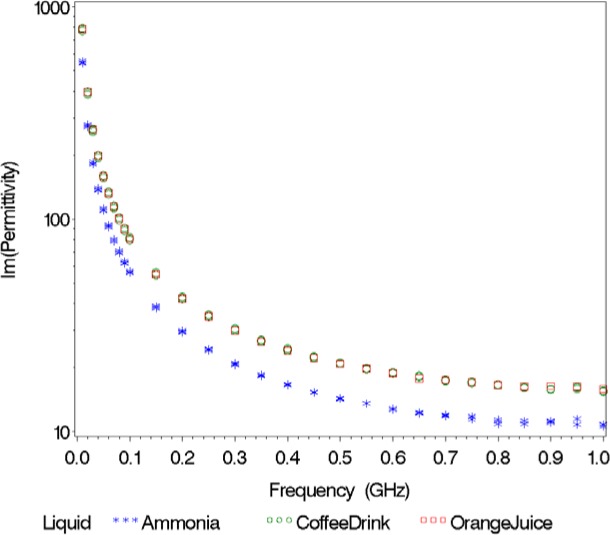
Imaginary part of relative permittivity versus frequency for orange juice (the unknown) and its nearest neighbors.

**Fig. 6:**
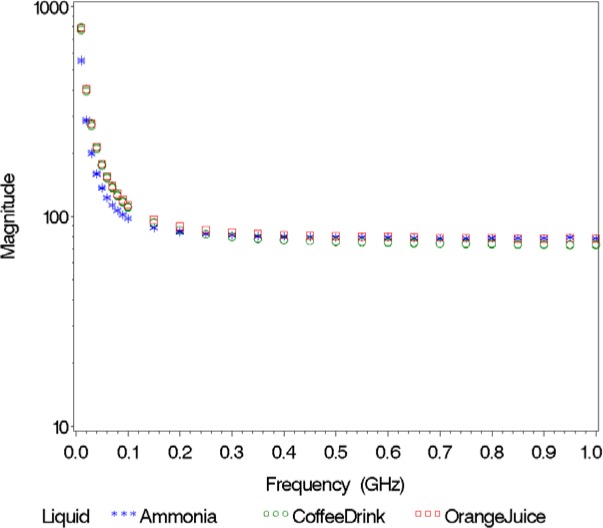
Magnitude of relative permittivity versus frequency for orange juice (the unknown) and its nearest neighbors.

Since the classification of orange juice is incorrect for *ε*′ and the combined *ε*′ and *ε*″ (we observed perfect classification for the combined *ε*′ and *ε*″ in Table 3 for Validation Study 1), we thought it would be useful to plot *ε*′, *ε*″, and magnitude versus frequency (Figs. 4–6). When implementing the nearest neighbor method, the resulting classification sometimes depends on the response considered. For instance, if we look at *ε*′, orange juice is similar to ammonia; however, if we examine *ε*″, orange juice is more like a coffee drink. The magnitude spectrum of orange juice is similar to the magnitude spectra of the coffee drink at low frequencies, but it is closer to the magnitude spectrum of ammonia at high frequencies.

**Fig. 7:**
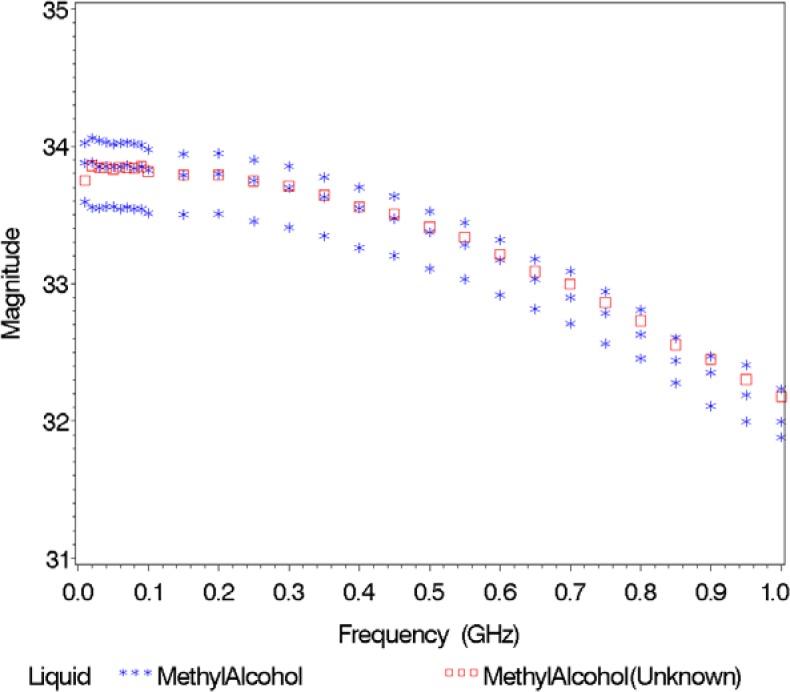
Magnitude of the relative permittivity versus frequency for the unknown (methyl alcohol) and its nearest neighbor (methyl alcohol).

In Fig. 7, we display the magnitude spectra versus frequency for the case where an unknown liquid in Validation Study 3, methyl alcohol, was also included in the database of liquids. Figures 4^7^ indicate that the classification of liquids is much easier if the liquid is contained in the database.

Although the main goal of our study is binary classification, it is interesting to examine the unknown liquids and their nearest neighbors in the database. In Table 5, we display the actual liquid and the liquid determined to be most similar to the unknown based on the nearest neighbor majority vote among the seven responses (from Table 4). The hazardous unknown liquids are: cognac, lighter fluid, lubricating oil, and methyl alcohol.

Unknown liquids that were not contained in the database (cognac, olive oil, energy drink, lubricating oil, and orange juice) were matched to a similar type of liquid in the database. All unknown liquids that were contained in the database were matched to the appropriate liquid.

### 3.3 Summary of Validation Studies

For Validation Study 1, two implementations of the nearest neighbor method based on the magnitude spectra and the combined real and imaginary spectra yielded misclassification rates of 0.0 (Table 3). This study suggests that our methods will be accurate if all unknown liquids of interest are represented in the primary database.

For Validation Study 2, misclassification error rates were no lower than 0.35 for all methods considered (Table 3). The study suggests that our classification methods based on only 20 liquids will not perform well in general. As a caveat, it is possible that a classification method based on a database with more liquids might perform well.

The relatively poor performance we observe in Validation Study 2 compared to Validation Study 3 is noteworthy. For Validation Study 3, the five unknown liquids not represented in the database were selected because they were similar to liquids in the database. Thus, the unknown liquids in Validation Study 3 were more similar to the liquids in the primary data base than the unknown liquids in Validation Study 2. Since the number of liquids considered in Validation Study 3 is small, it is difficult to reach general conclusions.

We stress that the spectra measured for the unknown liquids and the liquids in the primary database were performed under similar experimental conditions. If we had measured spectra in a less controlled environment, the performance of our classification methods might have been worse. Moreover, compared to the NIST measurement system, a realistic measurement system for practical application at airports or similar environments would surely yield less accurate spectral information. Hence, conclusions about the feasibility of a practical measurement system based on these preliminary measurements should be interpreted with caution.

## 4. Conclusions and Future Research

Although this initial feasibility study showed that identifying liquids with microwaves is possible, it was done by removing the liquid from the bottle and characterizing it in the shielded-open test fixture that was held at well-controlled environmental conditions. Although this was appropriate for the initial feasibility study, we now propose to investigate the much more interesting and practical problem of determining whether liquid identification is still possible when the unidentified liquid is in an unknown, unopened container at an unknown temperature. Because we have already developed a database of liquid dielectric spectra as well as the necessary classification techniques for identifying the liquids, we are in a good position to attack this more complicated problem.

Since one does not want to remove the liquid from the container to determine whether it is hazardous or not, we must now investigate the “signature” of the reflected and transmitted electromagnetic wave off of a liquid-filled container. However, instead of constructing a detection system for this purpose, we propose to first perform a series of systematic electromagnetic simulations that can calculate the reflected and transmitted wave off of the liquid-filled container. This “signature” will be a function of the electrical properties of the container and liquid, which we already have determined from our previous feasibility study, as well as the geometry of the bottle. Then, using the calculated reflection and transmission parameters, we will employ our previously-developed classification techniques to determine whether this is enough contrast to identify whether the liquid is hazardous or not.

This approach, which relies heavily on electromagnetic simulation, is powerful because of the flexibility it provides. We can easily change the electrical properties or temperature of the liquid and/or container, vary the geometry of the container, and even look at various ways of delivering the electromagnetic wave to the liquid-filled container. Through these simulations, we will be able to best identify which approach will lead to the most reliable detection method and then be in a good position to propose the construction of a practical hazardous liquid detection system.

## Figures and Tables

**Table 1:** Liquids selected for the validation studies.

Liquids for Validation Studies #1 & #2	“Unknown” Liquids for Validation Study #3
	
Acetone	Bottled Water
Ammonia	Cognac
Apple Juice	Contact Lens Solution
Baby Formula	Energy Drink
Bleach	Lighter Fluid
Bottled Water	Lubricating Oil
Coffee Drink	Methyl Alcohol
Contact Lens Solutions	Olive Oil
Corn Oil	Orange Juice
Cough Medicine	Vinegar
Liquid Fertilizer	
Hydrogen Peroxide	
Lighter Fluid	
Methyl Alcohol	
Motor Oil	
Shampoo	
Sunscreen	
Turpentine	
Vinegar	
Vodka	

**Table 2:** Summary of validation studies.

Validation Study	Data Set	Test Set
1	Primary database (59 spectra) contains two spectra of the same liquid as in the test set	Single spectrum from primary database
2	Primary database (57 spectra) does not contain spectra of the same liquid as in the test set	Three spectra of the same liquid from primary database
3	Primary database (60 spectra)	Ten new unknowns, five are represented in the primary database and five are not

**Table 3:** Overall (binary) misclassification error rates, true positive error rates, and false positive error rates for each response and the majority vote for Validation Studies 1 and 2.

	Validation Study 1			Validation Study 2		
		
Response	Overall Error Rate	True Positive	False Positive	Overall Error Rate	True Positive	False Positive
						
*ε*′	0.07	0.93	0.07	0.58	0.37	0.53
*ε*″	0.02	1.00	0.03	0.43	0.50	0.37
Magnitude of *ε*	0.00	1.00	0.00	0.45	0.30	0.20
Phase of *ε*	0.02	1.00	0.03	0.35	0.70	0.40
Loss tangent (*ε*′/*ε*″)	0.02	1.00	0.03	0.43	0.53	0.40
*ε*′ and *ε*″	0.00	1.00	0.00	0.45	0.37	0.20
Magnitude and Phase	0.02	1.00	0.03	0.37	0.70	0.40
Majority Vote	0.00	1.00	0.00	0.43	0.53	0.40

**Table 4:** Classification of unknown liquids (0=non-hazardous, 1=hazardous) based on the nearest neighbor method for each individual response. The majority vote and score are also displayed along with the true classification of the unknown liquid. Hazardous unknowns are highlighted in red while non-hazardous unknowns are highlighted in green. The liquids denoted by * were contained in the initial database of twenty liquids.

Unknown Liquid	*ε*′	*ε*″	Magnitude	Phase	Loss Tangent	*ε*′ & *ε*″	Magnitude & Phase	Majority Vote (Score)	Hazardous Determination
									
Vinegar*	0	0	0	0	0	0	0	0 (0/7=0)	N
Cognac	1	1	1	1	1	1	1	1 (7/7=1)	Y
Contact Solution*	1	0	0	0	0	0	0	0 (1/7=0.14)	N
Olive Oil	0	0	0	0	0	0	0	0 (0/7=0)	N
Lighter Fluid*	1	1	1	1	1	1	1	1 (7/7=1)	Y
Water*	0	0	0	0	0	0	0	0 (0/7=1)	N
Energy Drink	0	0	0	0	0	0	0	0 (0/7=1)	N
Lubricating Oil	1	1	1	1	1	1	1	1 (7/7=1)	Y
Methyl Alcohol*	1	1	1	1	1	1	1	1 (7/7=1)	Y
Orange Juice	1	0	0	0	0	1	0	0 (2/7=0.28)	N

**Table 5:** Nearest neighbor to the unknown liquid (majority vote from Table 4) and actual liquid. Hazardous liquids are highlighted in red while non-hazardous liquids are highlighted in green. The liquids denoted by * were contained in the initial database of twenty liquids.

Nearest Neighbor (majority vote)	Unknown Liquid
	
Vinegar	Vinegar*
Vodka	Cognac
Contact Solution	Contact Solution*
Corn Oil	Olive Oil
Lighter Fluid	Lighter Fluid*
Bottled Water	Bottled Water*
Apple Juice	Energy Drink
Motor Oil	Lubricating Oil
Methyl Alcohol	Methyl Alcohol*
Coffee Drink	Orange Juice
